# Emotion Regulation as a Predictor of Disordered Eating Symptoms in Young Female University Students

**DOI:** 10.3390/ejihpe15090171

**Published:** 2025-08-27

**Authors:** Marina Rojas-Valverde, Elena Felipe-Castaño

**Affiliations:** Department of Psychology and Anthropology, Faculty of Teacher Training, University of Extremadura, 10005 Caceres, Spain; mrojasva@alumnos.unex.es

**Keywords:** eating behaviour, emotion regulation, eating disorders, ROC curves

## Abstract

Eating disorders are characterised by concerns about food, body image, and weight control and/or reduction. They are more frequently described in women, and emotion regulation plays a central role in both their development and persistence. The aim of this study was to analyse the sensitivity and specificity of emotion regulation in predicting disordered eating symptoms in a sample of female university students. Non-probabilistic sampling was used to recruit 558 female university students, with a mean age of 20.63 years (*SD* = 1.88). An adaptation in Spanish of the Difficulties in Emotion Regulation Scale (DERS) and the Spanish version of the Eating Disorder Examination Questionnaire (S-EDE-Q) were administered. The findings suggested that scores related to emotion dysregulation, emotional rejection, and emotional interference may help distinguish women with disordered eating symptoms associated with food restriction and eating concerns. It is important to have sensitive tools that can identify at-risk populations as well as relevant psychological constructs linked to eating disorders when developing intervention programmes.

## 1. Introduction

Eating disorders (EDs) are characterised by body dissatisfaction and a fear of gaining weight, combined with physical and psychological maladjustment. These food-related symptoms are the visual manifestation of deep psychological distress, in which weight, body shape, and thinness become the main factors that influence an individual’s self-esteem and sense of self-worth (Arija-Val et al., 2022).

Risky and inappropriate eating behaviours have been growing alarmingly (González-Gómez et al., 2017). Common examples include binge eating, restrictive dieting, self-induced vomiting, the use of laxatives and/or diuretics, and fasting (Sysko et al., 2015). These behaviours often aim to control or reduce weight, and reflect an excessive preoccupation with food—whether it is eaten or not—and with body shape (Berengüí et al., 2016).

Disordered eating behaviours are considered harmful and may precede the onset of clinically diagnosed EDs such as bulimia nervosa or anorexia nervosa (Castro-López et al., 2021), as well as of more prevalent conditions in today’s world such as binge eating and obesity (Ruiz-Lázaro et al., 2010).

The factors that determine such eating behaviours and disorders are still not fully understood. Explanatory models tend to point to multifactorial and integrative frameworks, which include biological, psychological, and social factors. The most frequently studied factors include gender, eating styles, social norms, cognitive styles, and social media use.

It is generally agreed that women are ten times more likely than men to develop an eating disorder (American Psychiatric Association [APA], 2014), and this prevalence is still growing (Galmiche et al., 2019). In this context, research from different cultural environments identified that university students are a high-risk population for ED psychopathologies (Chang et al., 2015; Ko et al., 2015; Peláez-Fernández et al., 2013; Rø et al., 2015). Studies on EDs suggest that this heightened risk is associated with increased societal pressure on women to conform to unrealistic beauty standards, often perpetuated by the media and directly impacting body image (Venegas-Ayala & González-Ramírez, 2020).

In addition, cultural norms and the predominance of thin body ideals in mainstream and social media may trigger restrictive dieting (Grzymisławska et al., 2020; Sproesser et al., 2018; van Strien et al., 2014), or contribute to body dissatisfaction, which is recognised as a risk factor for eating disorders in women (Martínez-Gómez & Veiga Núñez, 2007; González et al., 2013; Rodríguez-Ruíz et al., 2013). According to Holland and Tiggemann (2017), there is a connection between the fitness-related content posted by women at risk of developing an ED (an estimated 17.5% according to the authors) and trends towards thinness, bulimia, and compulsive exercising.

One of the psychological factors that has been most widely studied is the difficulty in processing and/or regulating emotions (Corstorphine, 2006; Haynos & Fruzzetti, 2011; Lavender et al., 2015; Rowsell et al., 2016). Emotion regulation is defined as the awareness, understanding, and acceptance of one’s emotions; the ability to control impulsive behaviour and to engage in goal-directed behaviour during times of emotional distress; and the ability to use appropriate emotion regulation strategies in a flexible way to regulate emotional responses. Adaptive emotion regulation involves the ability to modify the emotional experience, allowing the individual to modulate how they experience emotions rather than suppressing them, effectively controlling the behaviour rather than the emotion (Thompson, 1994). The absence of one or more of these skills would therefore be an indicator of difficulties in emotion regulation (Melnick & Hinshaw, 2000).

There are many clinically relevant behaviours and constructs that are associated with emotion regulation deficits. The link between emotions and eating is complex, however. Some studies contend that negative emotions can trigger binge-eating episodes (Greeno et al., 2000; Nicholls et al., 2016), while others show that positive emotions can increase overall food intake (Leehr et al., 2015). Other researchers maintain that eating can be used as a means of regulating emotions (Stein et al., 2007).

People with EDs not only experience obsessive thoughts about thinness ideals, but they also have to contend with a series of poorly regulated and modulated emotional factors (Sawyer et al., 2024). Research into the relationship between emotion regulation and EDs may provide useful psychological insights for the development of public healthcare and preventive programmes. Furthermore, better understanding the sensitivity and specificity of an emotion regulation tool that predicts eating disorder risk in women could enable earlier identification of at-risk groups and inform public prevention strategies. However, no prior work has established culturally specific cut-off scores for such tools among Southern European student populations.

For these reasons, the aims of this study were twofold: first, to examine the relationship between difficulties in emotion regulation and disordered eating symptoms among female university students, and second, to assess the sensitivity and specificity of emotion regulation as a predictor of eating disorder risk in female university students. The study tested the following hypotheses: (a) There will be statistically significant relationships between emotion regulation and disordered eating symptoms, and (b) scores on emotion regulation scores will differentiate between participants at risk of developing an eating disorder and those who are not.

## 2. Materials and Methods

### 2.1. Participants

The sample consisted of 671 university students, of whom 561 (83.6%) were women and 110 (16.4%) were men, aged between 18 and 25 years (*M* = 20.66; *SD* = 1.86). The participants were recruited using non-probability convenience sampling from two public universities in Spain. At each university, four faculties were selected, and within each faculty, two class groups from each undergraduate programme were invited to participate.

Inclusion criteria specified that participants must be female, aged between 18 and 25 years, and have provided informed consent. Exclusion criteria included having a diagnosed eating disorder, currently receiving mental health treatment, or failing to meet one or more of the specified inclusion criteria.

Once the inclusion and exclusion criteria had been applied, the sample used for the study consisted of 558 female participants, aged between 18 and 25, with an average age of 20.63 (*SD* = 1.88). Male participants (*n* = 110) were excluded, as were three participants who reported having a diagnosed eating disorder. While no priori power analysis was performed, our post hoc sensitivity analysis confirmed that the sample size of 558 offered sufficient statistical power to detect meaningful effects and ensured the precise estimation of the discriminatory capacity of the tested variables via ROC analysis. Moreover, the sample size exceeded the recommended thresholds for reliable AUC analysis. According to Hajian-Tilaki (2014), at least 200 individuals per group are needed to achieve standard errors below 0.03 when groups are relatively balanced. This criterion was met in the present study, ensuring precise and stable estimates of diagnostic performance.

Most of the participants lived at home with their families (*n* = 350; 62.7%). The second largest group were living in shared flats or university halls of residence (*n* = 160; 28.7%). The last group consisted of participants who were living alone (*n* = 23; 4.1%) or with a partner (*n* =25; 4.5%). With regard to their studies, 58.3% (*n* = 325) were pursuing education-related degrees (Early Childhood and Primary Education or Social Education), while 41.7% (*n* = 233) were studying health sciences (Nursing or Occupational Therapy).

### 2.2. Instruments

Sociodemographic data included gender, age, the type of accommodation, degree course, and whether or not they had a diagnosed eating disorder or were receiving mental healthcare treatment.

The Spanish adaptation by Hervás and Jódar (2008) of the Difficulties in Emotion Regulation Scale (DERS, Gratz & Roemer, 2004) used for this study assesses difficulties in emotion regulation. The Spanish adaptation consists of 28 items rated on a five-point Likert-type scale ranging from 1 (almost never) to 5 (almost always). Higher scores indicate greater difficulties in emotion regulation. The Spanish adaptation includes five dimensions or subscales differing from the original six-factor model. Specifically, the Impulse and Strategies subscales were merged into a single factor, Emotional Dysregulation, and the remaining dimensions were relabelled to improve semantic clarity for the Spanish-speaking audience. The five-factor model was as follows: (1) Emotion Dysregulation (ED), which measures difficulties in controlling emotions and impulses, and in accessing emotion regulation strategies (combining the Impulse and Strategies subscales from the original English-language version by Gratz & Roemer, 2004); (2) Emotional Rejection (ER), which includes statements related to the acceptance or rejection of one’s own negative feelings of shame, anger, or guilt (original: Non-acceptance); (3) Emotional Interference (EINT), which measures difficulties in concentrating and accomplishing daily tasks when experiencing negative emotions (original: Goals); (4) Emotional Inattention (EI), which indicates a lack of awareness or inability to recognise or value one’s own emotions (original: Awareness); and (5) Emotional Confusion (EC), which consists of items that reflect the extent to which the individual understands and is clear about the emotions they are experiencing (original: Clarity). The Spanish adaptation has demonstrated good psychometric properties in Spanish university populations, supporting its use in the present study (Hervás & Jódar, 2008). The internal consistency values, calculated using Cronbach’s alpha (α) taken from the participant sample, can be seen in Table 1.

The Eating Disorder Examination Questionnaire—Spanish version, S-EDE-Q (Peláez-Fernández et al., 2012), which was used for this study, is the adaptation into Spanish of the Eating Disorder Examination Questionnaire (EDE-Q, Fairburn & Beglin, 1994), which, in turn, is an adaptation of the Eating Disorder Examination interview (EDE; Fairburn & Cooper, 1993). The EDE-Q is one of the most widely used measures for screening and assessing EDs in community populations. The Spanish adaptation is a 36-item self-report questionnaire that assesses dietary restriction, behaviours, attitudes, and feelings related to eating, body shape, weight, etc., over the past 28 days, using a seven-point Likert-type response scale ranging from 0 (never) to 6 (every day). The questionnaire is categorised into four subscales: (1) Dietary Restriction, which refers to actions aimed at reducing food intake to prevent it affecting a person’s shape and/or weight; (2) Eating Concern, related to the cognitive aspect of preoccupation with food intake, e.g., the emotions experienced when thinking about eating, or the shame associated with doing so; (3) Weight Concern, referring to the interference in daily life caused by excessive preoccupation with weight and the fear of being fat; and (4) Shape Concern, which reflects concerns about body shape and a desire to have physical features one does not possess, e.g., a flat stomach.

The S-EDE-Q allows the identification of individuals who may be at risk of developing EDs. To this end, a mean score of ≥4 points is considered to indicate potential clinical significance (Peláez-Fernández et al., 2012). The internal consistency values for each of the scales, calculated using Cronbach’s alpha (α), can be seen in Table 1.

### 2.3. Procedure

First, an application for approval for the study was sent to the University’s Bioethics Committee (Code 142/2023), including an informed consent form. The application was drafted in accordance with the recommendations contained in the Declaration of Helsinki, in compliance with the rules and regulations regarding personal data protection (Organic Law 3/2018 dated 5 December). Due to the sensitive nature of the content investigated, the informed consent form stated that participants could withdraw from the study at any time, particularly if they experienced emotional discomfort or distress related to the self-report measures. In such cases, information about the psychological counselling services available through the university was provided.

Data collection was carried out within class groups. Having obtained permission from the teachers of the selected class groups, the questionnaire was administered in the classroom using pencil and paper. Instructions were given at the beginning of the session pointing out the voluntary nature of participation and the anonymity and confidentiality of the responses. The entire process took approximately 20–25 min. Although the research focused specifically on female participants, data were gathered from all the students present, both male and female, in order to avoid disrupting the assessment process. Following collection, the data were coded in an SPSS file for subsequent analysis.

### 2.4. Data Analysis

Normality was assessed using the Kolmogorov–Smirnov (K–S Test), which indicated that all of the variables were normally distributed. Internal consistency was examined using Cronbach’s alpha with a 95% CI, and descriptive analyses were conducted for the questionnaire scales' scores. Parametric statistical tests were used for data analysis.

To examine the relationship between emotion regulation and disordered eating symptoms, Pearson’s correlation coefficient was used. To assess the sensitivity and specificity of the scores in the difficulties in emotion regulation scale in predicting eating disorder scores, a logistic regression analysis was conducted using receiver operating characteristic curves (ROC). For this purpose, 95% confidence intervals were calculated in conjunction with analysing the ROC curve. This method allows the diagnostic accuracy of tests with continuous measurement scales to be determined. In addition, the Youden Index was calculated. The Youden Index is used for three specific purposes: to identify the optimal cut-off point at which sensitivity and specificity are maximised; to assess the discriminative ability of the test; and to compare the predictive capacity of two or more diagnostic tools (Cerdá & Cifuentes, 2012). This approach is widely accepted in the biomedical literature for diagnostic validation studies and is recommended when the goal is to achieve good overall discrimination without explicitly prioritising sensitivity or specificity (Fluss et al., 2005; Youden, 1950).

Data coding and analysis were carried out using the statistical software package SPSS (version 25.0). Comparisons between ROC curves were conducted using MedCalc (https://www.medcalc.org/) (accessed on 2 May 2025). A 5% margin of error was assumed for hypothesis testing, with the significance level set at *p* ≤ 0.05.

## 3. Results

Table 1 presents descriptive statistics for the scores obtained on the questionnaire scales and dimensions, along with internal consistency values.

**Table 1:** Descriptive statistics and internal consistency values. Scale scores of the S-EDE-Q and DERS questionnaires.

S-EDE-Q	M	SD	Range	Minimum	Maximum	Cronbach’s α [95% CI]
Dietary Restriction (DR)	1.36	1.47	6.00	0.00	6.00	0.876 [0.859–0.892]
Eating Concern (EaC)	1.02	1.21	5.40	0.00	5.40	0.823 [0.799–0.845]
Weight Concern (WC)	1.88	1.59	6.00	0.00	6.00	0.871 [0.853–0.887]
Shape Concern (SC)	2.05	1.65	6.00	0.00	6.00	0.933 [0.924–0.941]
Total Score (TS)	6.33	5.48	22.15	0.00	22.15	0.963 [0.959–0.968]
DERS						
Emotion Dysregulation (ED)	19.97	8.22	36	9	45	0.911 [0.898–0.924]
Emotional Rejection (ER)	15.16	6.81	28	7	35	0.904 [0.889–0.918]
Emotional Interference (EINT)	12.38	4.11	16	4	20	0.874 [0.852–0.893]
Emotional Inattention (EI)	14.27	3.52	16	4	20	0.829 [0.800–0.855]
Emotional Confusion (EMC)	14.39	3.44	16	4	20	0.825 [0.835–0.876]

Statistically significant correlations were found between all dimensions of the Difficulties in Emotion Regulation Scale and the scales of the Eating Disorder Questionnaire. Significant positive correlations were observed between Dietary Restriction, Weight Concern, Eating Concern, and Shape Concern, and Emotion Dysregulation, Emotional Rejection, and Emotional Interference, while significant negative correlations were found with Emotional Inattention and Emotional Confusion (see Table 2).

The percentage and number of participants with possible clinical significance, based on a score of four or higher on the S-EDE-Q scales (Peláez-Fernández et al., 2012, 2013), were as follows: 8.6% (*n* = 48) for the Dietary Restriction scale; 4.5% (*n* = 25) for Eating Concern; 13.8% (*n* = 77) for Weight Concern; and 16.5% (*n* = 92) for Shape Concern. A total of 19.2% (*n* = 107) of the participants obtained scores that were of potential clinical significance on at least one of the scales on the eating disorder questionnaire. Based on these results, participants were classified as either clinically significant or non-significant according to their total S-EDE-Q scores.

**Figure 1:**
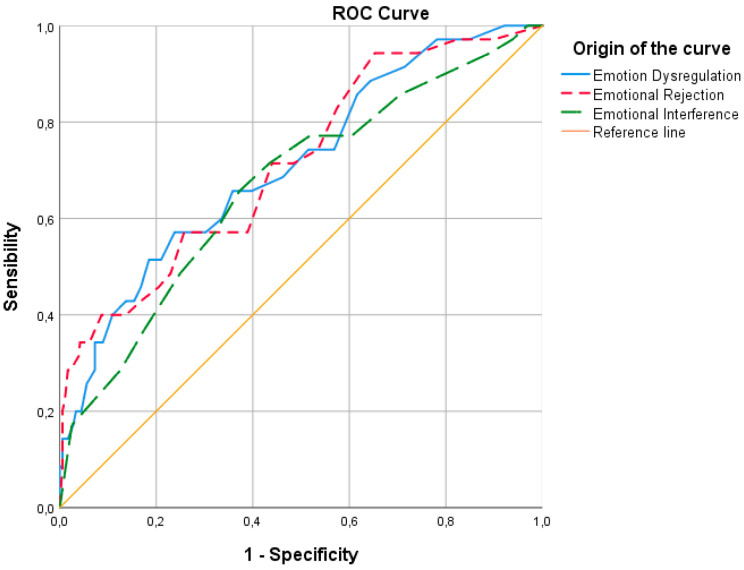
ROC curve for Emotion Dysregulation, Emotional Rejection, and Emotional Interference as predictors of the Dietary Restriction subscale.

In the non-parametric ROC curve analysis for the Dietary Restriction subscale (Figure 1), Emotion Dysregulation (ED; AUC = 0.710, *p* ≤ 0.001, 95% CI [0.618–0.802]) and Emotional Rejection (ER; AUC = 0.714, *p* ≤ 0.001, 95% CI [0.622–0.806]) demonstrated moderate discriminatory capacity. Emotional Interference (EINT; AUC = 0.669, *p* ≤ 0.001, 95% CI [0.572–0.767]) demonstrated poor discriminatory capacity. Inattention (EI) did not significantly discriminate between groups (AUC = 0.477, *p* = 0.654, 95% CI [0.365–0.589]), and Emotional Confusion (EC) showed discriminatory performance below chance level (AUC = 0.354, *p* = 0.004, 95% CI [0.253–0.455]).

These results indicate that the Emotional Rejection scale was the most effective in distinguishing individuals with high levels of Dietary Restriction, as it yielded the highest AUC value. Emotion Dysregulation demonstrated acceptable accuracy, with the AUC reflecting fair diagnostic performance according to established criteria (Çorbacıoğlu & Aksel, 2023). Emotional Interference showed lower accuracy, indicating poor diagnostic performance. The remaining scales did not yield statistically significant results and were therefore excluded from the ROC curve analysis (see Figure 1).

**Figure 2:**
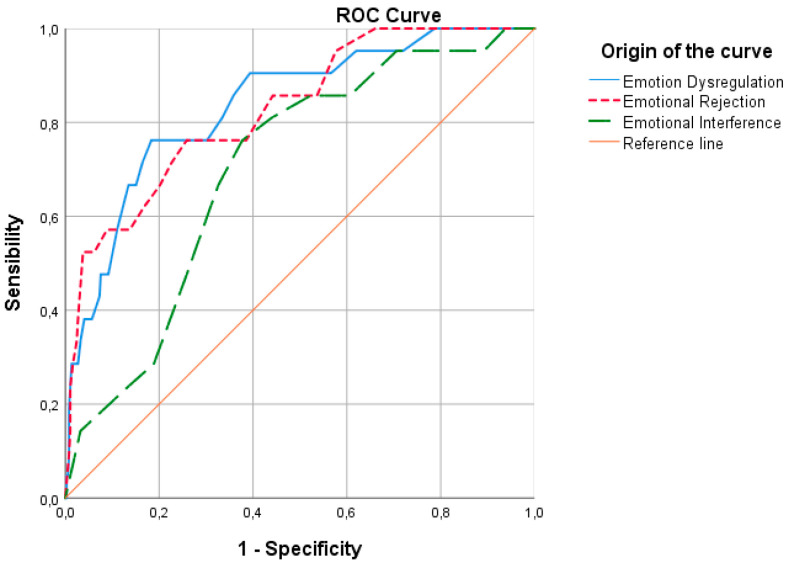
ROC curve for Emotion Dysregulation, Emotional Rejection, and Emotional Interference as predictors of the Eating Concern subscale.

In the non-parametric ROC curve analysis for the Eating Concern subscale (Figure 2), the area under the curve (AUC) was 0.837 (*p* ≤ 0.001; 95% CI [0.749–0.925]) for Emotion Dysregulation (ED), 0.827 (*p* ≤ 0.001; 95% CI [0.737–0.918]) for Emotional Rejection (ER), and 0.702 (*p* = 0.002; 95% CI [0.601–0.803]) for Emotional Interference (EINT). The AUC was 0.404 (*p* = 0.010; 95% CI [0.330–0.478]) for Emotional Inattention (EI) and 0.341 (*p* ≤ 0.001; 95% CI [0.270–0.411]) for Emotional Confusion (EC). The highest discriminatory capacity was observed for Emotion Dysregulation, followed by Emotional Rejection and Emotional Interference. According to Çorbacıoğlu and Aksel (2023), these AUC values indicate good discriminative power.

**Figure 3:**
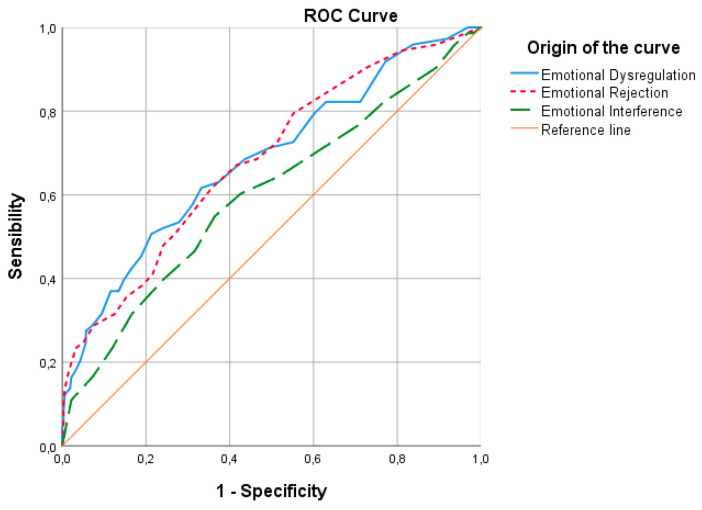
ROC curve for Emotion Dysregulation, Emotional Rejection, and Emotional Interference as predictors of the Weight Concern subscale.

In the non-parametric analysis of the ROC curves for the Weight Concern subscale (see Figure 3), the area under the curve (AUC) was 0.684 (*p* ≤ 0.001; 95% CI [0.613–0.755]) for Emotion Dysregulation (ED); 0.683 (*p* ≤ 0.001; 95% CI [0.614–0.752]) for Emotional Rejection (ER); and 0.599 (*p* = 0.039; 95% CI [0.523–0.675]) for Emotional Interference (EINT). The AUC for Emotional Inattention (EI) was 0.374 (*p* = 0.052; 95% CI [0.246–0.503]) and for Emotional Confusion (EC) it was 0.222 (*p* ≤ 0.001; 95% CI [0.115–0.328]). The Emotion Dysregulation scale demonstrated the highest ability to distinguish participants with elevated Weight Concern scores, followed by the Emotional Rejection and Emotional Interference scales. However, according to Çorbacıoğlu and Aksel (2023), the AUC values in this study indicate poor discriminatory power, suggesting that DERS scales did not adequately predict weight concerns in this sample.

**Figure 4:**
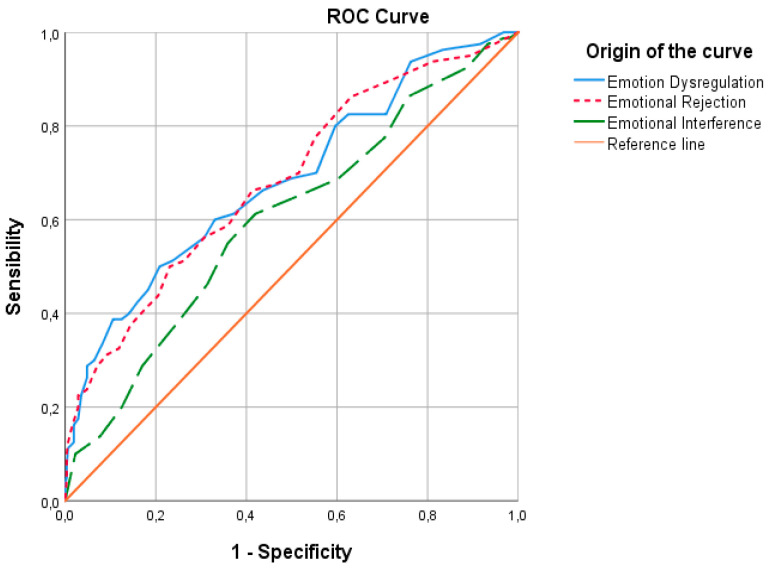
ROC curve for Emotion Dysregulation, Emotional Rejection, and Emotional Interference as predictors of the Shape Concern subscale.

In the Shape Concern subscale (see Figure 4), the area under the curve (AUC) was 0.685 (*p* ≤ 0.001; 95% CI [0.617–0.754]) for Emotion Dysregulation (ED), 0.683 (*p* ≤ 0.001; 95% CI [0.616–0.751]) for Emotional Rejection (ER), and 0.601 (*p* = 0.005; 95% *CI* [0.530–0.672]) for Emotional Interference (EINT). Emotional Inattention (EI) and Emotional Clarity (EC) showed lower AUC values of 0.416 (*p* = 0.021; 95% CI [0.343–0.490]) and 0.329 (*p* ≤ 0.001; 95% CI [0.258–0.400]), respectively. Emotion Dysregulation showed the highest discriminative capacity, followed by Emotional Rejection and Emotional Interference. However, according to Çorbacıoğlu and Aksel (2023), these AUC values are considered poor, indicating that DERS scales did not adequately predict shape concerns in this sample.

Table 3 presents the cut-off points based on the sensitivity and specificity values for each scale and the Youden Index. This index suggests that the scores on the Emotion Dysregulation, Emotional Rejection and Emotional Interference scales were the most useful for distinguishing participants with Eating Concern issues.

The DeLong test was used to compare the ROC curves obtained for each S scale (DeLong et al., 1988). Significant differences were found between the AUCs of Emotion Dysregulation and Emotional Interference for the Eating Concern scale (DEA = 0.135; SE = 0.0534; 95% CI [0.0309–0.240]; *Z* = 2.538; *p* = 0.0111); the Weight Concern scale (DEA = 0.0847; SE = 0.0333; 95% CI [0.0195–0.150]; *Z* = 2.548; *p* = 0.0108); and the Shape Concern scale (DEA = 0.0845; SE = 0.0325; 95% CI [0.0207–0.148]; *Z* = 2.598; *p* = 0.0094). Significant differences were also observed between the AUCs of Emotional Rejection and Emotional Interference for the Weight Concern scale (DEA = 0.0838; SE = 0.0395; 95% CI [0.00625–0.161]; *Z* = 2.118; *p* = 0.0342) and the Shape Concern scale (DEA = 0.0862; SE = 0.0388; 95% CI [0.00658–0.159]; *Z* = 2.129; *p* = 0.0332).

Given the characteristics of the assessment tools and the non-clinical nature of the sample, we focused on the cut-off scores that prioritised sensitivity over specificity, i.e., scores more effective at identifying individuals at greater risk of developing EDs, rather than confirming their presence.

## 4. Discussion

The main aim of the study was to analyse the relationship between emotion regulation and disordered eating symptoms, and to examine the sensitivity and specificity of emotion regulation scores in predicting the presence of eating disorders symptoms in a sample of female university students.

Regarding the first hypothesis, the findings confirmed the expected outcome. Statistically significant correlations were observed between emotion regulation and EDs. Previous research similarly links EDs to poor emotion regulation (da Luz et al., 2023). DERS subscales have been shown to correlate significantly with the global S-EDE-Q (Monell et al., 2015). In individuals with EDs, emotional rejection and emotion dysregulation are the most affected dimensions compared with healthy controls and normative population benchmarks (Calvo Sagardoy et al., 2014). Lower scores on the Difficulties in Emotion Regulation Scale (DERS) are associated with reduced ED symptoms; though not with body mass index in anorexia nervosa (Haynos et al., 2014; Racine & Wildes, 2013). Certain emotion regulation difficulties also relate to specific ED behaviours: self-induced vomiting and higher diuretic use to higher Emotional Rejection, and binge eating to Emotion Dysregulation, Emotional Rejection, and Emotional Interference (Calvo Sagardoy et al., 2014).

Regarding the second hypothesis, scores for Emotion Dysregulation, Emotional Rejection and Emotional Interference helped distinguish women with EDs characterised by dietary restriction and eating concerns. They were less useful for weight and body shape concerns, with the strongest associations observed for eating concern-related behaviours. The proposed cut-off points remain preliminary and require replication before widespread adoption and clinical use.

Food restriction is commonly observed in patients diagnosed with anorexia nervosa. It serves both as a strategy for weight loss and as a maladaptive way to reduce negative emotions. In acute anorexia, a specific association has been identified between body weight and emotion regulation: lower BMI has been linked to fewer reported difficulties in emotion regulation (Brockmeyer et al., 2012). This suggests that dietary restriction could temporarily reduce negative affect through a mechanism of negative reinforcement (Espeset et al., 2011; Fox & Froom, 2009; Gilboa-Schechtman et al., 2006).

Conversely, improvements in affective functioning are associated with reduced ED risk (Preyde et al., 2016). Eating concern behaviours may act as coping strategies for negative emotions, persisting when individuals struggle to resist emotionally-rewarding actions. This pattern can hinder weight loss in obesity and promote weight loss in EDs (Segura-Serralta et al., 2019).

Better emotion recognition and regulation, along with stronger beliefs in the effectiveness of regulation strategies, are linked to fewer cognitive distortions about eating, weight, and body image (Brown et al., 2020; Monell et al., 2018; Sawyer et al., 2024).

While weight and shape concerns remain clinically relevant, they may be less sensitive indicators of affective processes. Emotion dysregulation appears more strongly tied to body dissatisfaction, body image overvaluation, and eating-related impulsivity (Lavender et al., 2015).

These findings highlight the importance of addressing emotion regulation in ED treatment. Patients often see managing emotions as equally important as addressing eating behaviours. Interventions combining cognitive behavioural therapy, interpersonal therapy, and dialectical behaviour therapy (involving relaxation training and eating behaviour awareness) have shown positive results. They reduce eating concerns, body image issues, and dietary restriction in both outpatient (Clyne et al., 2010) and inpatient settings (Brown et al., 2020). Treatment models that integrate emotion regulation show particular promise (Roos et al., 2021; Sala et al., 2023). In contrast, interventions that neglect emotional factors and rely on rigid, coercive control may yield only temporary improvements, with relapse common once monitoring ends (Calvo Sagardoy et al., 2014).

These interventions have demonstrated positive effects in reducing eating concerns, body image concerns and dietary restriction in outpatients with EDs (Clyne et al., 2010) as well as in inpatients (Brown et al., 2020). Their importance is also evident in ED intervention models that include emotion regulation as a key component of the treatment (Roos et al., 2021; Sala et al., 2023). Conversely, when emotional aspects are neglected during treatment, the outcomes of rigid and coercive interventions (featuring surveillance and control) tend to be misinterpreted as successful, when, in fact, the benefits are often temporary as patients often relapse once ‘controls’ are no longer present (Calvo Sagardoy et al., 2014).

This study has several limitations that should be considered when interpreting the findings. Its cross-sectional design prevents establishing causal relationships between variables, and reliance on self-report measures may have introduced biases such as social desirability and response sincerity. Although the sample was large, it comprised only women aged 18 to 25, a period when EDs may already be well-established, potentially reflecting entrenched behavioural patterns. As EDs often emerge during adolescence (Trompeter et al., 2022)—a stage marked by substantial physical and psychological changes and heightened body dissatisfaction (Nelson et al., 2021)—the participants’ age may have influenced their S-EDE-Q scores.

Future research should use longitudinal designs to better assess the temporal and potentially causal relationship between emotion regulation difficulties and the development of disordered eating symptoms. To address the limitations of self-report measures, complementary methods, such as clinical interviews or behavioural tasks could be incorporated. Replicating the study with a clinical sample diagnosed with EDs would help evaluate the diagnostic accuracy and generalisability of the proposed cut-off points (e.g., calibration curve or decision curve analysis). Potential confounders—such as social media influence, body mass index, or anxiety and depressive symptoms—should be incorporated to clarify their role in the relationship between emotion regulation and eating disorder risk. Finally, including male participants and extending the age range to adolescents and preadolescents would enable a more comprehensive assessment of these thresholds across developmental stages and demographic groups.

## 5. Conclusions

Emotional problems, such as emotion dysregulation, are recognised as predisposing factors in the development of eating disorders (Cooper et al., 2004; Fox & Froom, 2009; Vögele & Gibson, 2010). Sensitive instruments for detecting at-risk behaviours in young women enable early identification of EDs and timely intervention, thereby improving prognosis (Fukutomi et al., 2020; McClelland et al., 2018). Identifying explanatory variables that clarify risk factors and shed light on emotional biases may further advance our understanding of eating disorders and enhance the effectiveness of intervention strategies. Finally, reliable, sensitive assessment tools for detecting risk in non-clinical populations are essential for strengthening prevention programmes.

## Figures and Tables

**Table 2:** Correlation analysis. DERS and S-EDE-Q scales.

DERS	S-EDE-Q				
	DR	EaC	WC	SC	TS
ED	0.291 **	0.444 **	0.386 **	0.377 **	0.402 **
ER	0.283 **	0.420 **	0.366 **	0.352 **	0.381 **
EINT	0.169 **	0.266 **	0.252 **	0.235 **	0.248 **
EI	−0.100 *	−0.193 **	−0.191 **	−0.176 **	−0.178 **
EC	−0.238 **	−0.396 **	−0.344 **	−0.352 **	−0.358 **

Note: DR: Dietary Restriction; EaC: Eating Concern; WC: Weight Concern; SC: Shape Concern; TS: Total Score; ED: Emotion Dysregulation; ER: Emotional Rejection; EINT: Emotional Interference; EI Emotional Inattention; and EC: Emotional Confusion. ** *p* ≤ 0.001; * *p* ≤ 0.01.

**Table 3:** Cut-off points based on sensitivity and specificity values for Emotion Dysregulation, Emotional Rejection, and Emotional Interference high-risk predictors on the subscales of Dietary Restriction, Eating Concern, Weight Concern, and Shape Concern.

Predictor	Criteria	Cut-Off Point	Sensitivity	95% CI	1—Specificity	95% CI	Youden Index
Emotion Dysregulation	Dietary Restriction	16.5 **	0.743	0.567–0.875	0.569	0.379–0.484	0.174
		17.5	0.743		0.515		0.227
		18.5 *	0.686		0.462		0.224
	Eating Concern	20.5 **	0.857	0.636–0.969	0.358	0.590–0.690	0.499
		21.5 *	0.810		0.334		0.475
	Weight Concern	18.5 **	0.685	0.565–0.788	0.436	0.507–0.619	0.249
	Shape Concern	17.5 **	0.688	0.574–0.786	0.497	0.446–0.560	0.191
		18.5 *	0.663		0.436		0.227
Emotional Rejection	Dietary Restriction	13.5 **	0.714	0.537–0.853	0.485	0.462–0.568	0.230
		14.5 *	0.614		0.440		0.275
	Eating Concern	14.5 **	0.857	0.636–0.969	0.442	0.505–0.609	0.415
	Weight Concern	13.5 **	0.685	0.565–0.788	0.464	0.479–0.591	0.221
	Shape Concern	12.5 **	0.700	0.589–0.797	0.516	0.427–0.541	0.184
		13.5 *	0.675		0.462		0.213
Emotional Interference	Dietary Restriction	12.5 **	0.714	0.537–0.853	0.434	0.512–0.617	0.280
		13.5 *	0.657		0.373		0.285
	Eating Concern	13.5 **	0.762	0.528–0.917	0.377	0.571–0.672	0.385
	Weight Concern	12.5 **	0.603	0.481–0.715	0.426	0.517–0.628	0.176
		13.5 *	0.548		0.364		0.184
	Shape Concern	12.5 **	0.613	0.497–0.719	0.420	0.523–0.635	0.193
		13.5 *	0.550		0.329		0.191

Note: * Score maximises sensitivity; ** Score maximises both sensitivity and specificity.

## Data Availability

The raw data (de-identified data and analysis scripts) supporting the conclusions of this article will be made available by the authors on request.
